# Carbapenem-resistant Enterobacterales bacteraemia at a tertiary hospital: A five-year review

**DOI:** 10.4102/sajid.v41i1.765

**Published:** 2026-01-09

**Authors:** Dewald Marais, Bonita van der Westhuizen, Claire L. Barrett, Samantha Potgieter

**Affiliations:** 1 Department of Internal Medicine, Faculty of Health Sciences, University of the Free State, Bloemfontein, South Africa; 2 Department of Medical Microbiology, Faculty of Health Sciences, University of the Free State, Bloemfontein, South Africa; 3 Department of Medical Microbiology, National Health Laboratory Services, Universitas Hospital, Bloemfontein, South Africa; 4 Research and Development Unit, Faculty of Health Sciences, University of the Free State, Bloemfontein, South Africa

**Keywords:** Carbapenem-resistant Enterobacterales (CRE), bacteraemia, *Klebsiella pneumoniae*, antimicrobial resistance, under-resourced healthcare setting

## Abstract

**Background:**

Carbapenem-resistant Enterobacterales (CRE) pose a critical threat to public health, marked by limited therapeutic options, high mortality rates and significant pressure on healthcare systems. Despite the growing global burden, our region remains under-represented in national surveillance efforts, with a notable absence of local data.

**Objectives:**

This study aims to describe the epidemiological, clinical and microbiological characteristics, as well as patient outcomes, of CRE bacteraemia at Universitas Academic Hospital in Bloemfontein, South Africa, over a 5-year period.

**Method:**

A retrospective file review was performed for all adult in-patients with confirmed CRE bacteraemia admitted between 2019 and 2023. Data collected included patient demographics, comorbidities and clinical data pertaining to admission, microbial characteristics and clinical outcomes.

**Results:**

Ninety-four episodes of CRE bacteraemia were identified in 88 patients. Prior antibiotic exposure was present in 90.9%, while 79.5% had comorbidities and 61.4% acute renal impairment. *Klebsiella pneumoniae* (84%) and *Enterobacter cloacae* (9.6%) were the predominant organisms cultured, with oxacillinase-48 (OXA-48) (78.4%) and New Delhi metallo-β-lactamase (NDM) (6.7%) being the most common carbapenemase genes detected. Only 13.8% of OXA-48-positive episodes received recommended first-line antibiotics. In-hospital mortality reached 56.8%, with immunosuppressive therapy significantly associated with death (*p* = 0.0165).

**Conclusion:**

Mortality in our setting was substantially higher than national and international reports. Suboptimal treatment and limited access to effective antimicrobials likely contributed to these poor outcomes.

**Contribution:**

This is the first outcome-focused CRE study in this region, highlighting an urgent need for improved diagnostic capacity, antimicrobial access and targeted intervention strategies in under-resourced healthcare settings.

## Introduction

Antimicrobial resistance (AMR) is a growing global health concern and, in 2021, was linked to 1.14 million deaths worldwide, with projections estimating this figure could rise up to 10 million deaths annually by 2050.^1^ Among the most concerning contributors are carbapenem-resistant Enterobacterales (CRE), which are responsible for an estimated 3.1 million infections annually and ranking among the top six pathogens associated with AMR-related mortality.^2,3^ First identified in Japan during the 1980s, CRE have since spread worldwide, largely driven by the widespread and often unregulated use of antibiotics.^4^ In South Africa, CRE bacteraemia cases rose by 57% between 2018 and 2019, with notable regional variation observed during the 2019–2020 period.^5,6^ This escalating crisis underscores the urgent need for coordinated action to curb the spread of resistance and preserve the effectiveness of existing antimicrobials.

Enterobacterales, a group of Gram-negative, rod-shaped bacteria, are commonly implicated in both healthcare-associated and community-acquired infections.^7^ Carbapenem-resistant Enterobacterales are defined by their resistance to at least one carbapenem antibiotic, often mediated through the production of carbapenemase enzymes such as *Klebsiella pneumoniae* carbapenemase (KPC), New Delhi metallo-β-lactamase (NDM), oxacillinase-48 (OXA-48), Guiana extended-spectrum β-lactamase (GES) and Verona integron-encoded metallo-β-lactamase (VIM).^8^ These enzymes were first identified in South Africa during 2011–2012.^6^ Given the high mortality associated with CRE bacteraemia and the limited treatment options, understanding the microbial and clinical characteristics, as well as the outcomes, is vital for guiding infection prevention and antimicrobial stewardship efforts.^6,9^

Despite national surveillance efforts, data from the Free State Province remain sparse. The most recent national surveillance study by the Group for Enteric, Respiratory and Meningeal Disease Surveillance in South Africa (GERMS-SA) (2019–2020) included only 41 Free State cases out of 2144 nationally, limiting the applicability of findings to this region.^6^ This study aims to address that gap by describing the patient profile, microbial patterns and the outcomes of CRE bacteraemia cases at Universitas Academic Hospital in Bloemfontein from 2019 to 2023.

## Research methods and design

### Study design

A retrospective descriptive study was performed.

### Setting

This study was conducted at Universitas Academic Hospital in Bloemfontein, located in Free State Province of South Africa. The hospital is a 636-bed public healthcare facility that functions as a tertiary referral centre for the Free State and Northern Cape provinces. It houses the only Clinical Haematology Unit in Central South Africa and offers specialised intensive care across various medical and surgical disciplines. In addition, the hospital provides sub-specialist services to Lesotho, a sovereign enclave within South Africa.

### Study population

Results of all patients aged 13 years and older with CRE bacteraemia who were admitted to adult wards between 01 January 2019 and 31 December 2023 were included.

Carbapenem-resistant Enterobacterales bacteraemia was defined in line with the criteria established by the Centers for Disease Control and Prevention (CDC). A case was identified when a patient had at least one positive blood culture for an Enterobacterales species resistant to at least one carbapenem antibiotic – ertapenem, meropenem, imipenem or doripenem. This included organisms that produced carbapenemase enzymes. For organisms such as *Proteus* spp., *Morganella* spp. and *Providencia* spp., which inherently exhibit elevated minimum inhibitory concentrations (MICs) to imipenem, susceptibility to ertapenem, meropenem and doripenem was used to determine whether the isolate met the definition of a CRE.^8^

### Data collection

Cases were identified through the Infection Prevention and Control (IPC) unit, which receives CRE-positive blood culture notifications from the Divisions of Medical Microbiology (National Health Laboratory Services, NHLS) and Infectious Diseases, Free State Department of Health. These cases are recorded on an internal electronic alert organism spreadsheet. Eligible cases were captured using REDCap data collection software, with identifiers (name, surname, hospital number) entered on a separate enrolment sheet, distinct from the main data collection tool. Each case was assigned a unique study number to ensure pseudo-anonymisation. The enrolment sheet linked each CRE case to the corresponding clinical, pharmacy and laboratory data.

Additional positive blood cultures for the same CRE organism in a single patient were counted as a new episode if cultured more than 21 days after the preceding culture. For patients in whom more than one CRE organism was cultured, separate episodes were entered under the same participant.

### Variables

Comprehensive demographic and clinical data were collected. Numerical variables included age, length of stay at the time of positive blood culture and total hospital stay. Categorical variables included sex, comorbidities, recent surgeries, antibiotic use within 6 months, current exposure to immunosuppressive therapy, ward location and admissions in the preceding 90 days. Microbiological data encompassed organism type, susceptibility profile, resistance genes and concurrent sites of CRE isolation. The Vitek2 system was used for phenotypic microbial identification and antimicrobial susceptibility testing. The antimicrobial susceptibility testing results were interpreted in accordance with the Clinical and Laboratory Standards Institute (CLSI) guidelines, categorising isolates as susceptible, intermediate or resistant, based on established breakpoints. During the study period, the E-test method was not locally used to determine carbapenem MICs, nor was the MIC result reported to the treating clinician. Isolates were referred to the National Institute for Communicable Diseases for determination of resistance genes using molecular methods. Antimicrobial treatment received was recorded from electronic pharmacy records. During the study period, it was local practice to prescribe carbapenem monotherapy for isolates demonstrating full susceptibility to a carbapenem, based on Vitek2 system results. For isolates where no carbapenem was fully susceptible, a combination of a carbapenem and colistin was prescribed. Ceftazidime–avibactam became available towards the end of the study period, and, per local practice, patients who were critically ill, neutropenic or not responding to alternative regimens were changed to this drug once OXA-48 enzyme production was confirmed. Outcomes recorded were total hospital stay and in-hospital mortality. Specific definitions of variables can be found in Appendix 1.

### Data analysis

Descriptive statistics were calculated, including frequencies and percentages for categorical data, and medians with interquartile ranges for numerical data (since the distribution was skewed). The number of days between episodes was assessed using the Wilcoxon signed-rank test.

The analysis was conducted by the Department of Biostatistics, University of the Free State, using SAS/STAT software, Version 9.4 of the SAS System for Windows (Copyright © 2013 SAS Institute Inc.). The association between clinical outcomes and factors such as sex, underlying malignancy, HIV status, comorbid conditions, current use of immunosuppressive therapy, documented antibiotic exposure within the past 6 months, recent surgical procedures and hospitalisations within the last 90 days was examined using the chi-square test or Fisher’s exact test (for sparse data) for categorical variables. A *p* < 0.05 was considered to be statistically significant.

### Ethical considerations

Ethical approval was obtained from the Health Sciences Research Ethics Committee (HSREC) of the University of the Free State (No. UFS-HSD2024/0277/1806). Data collection began only after permissions were granted by the Free State Department of Health, the Head of Internal Medicine at Universitas Academic Hospital and the NHLS Business Manager.

All data were collected retrospectively and managed in line with ethical standards to ensure confidentiality. The enrolment sheet containing personal identifiers was securely stored on REDCap, accessible only to the principal researcher, and will be retained for 5 years. No identifying data were included in the main dataset, in compliance with the *Protection of Personal Information Act* (*POPIA*, 2013). As the study used anonymised retrospective data with no patient contact, informed consent was not required.

## Results

A total of 88 patients with CRE bacteraemia were reported to the IPC department during the 5-year study period. Of these, six patients had a second episode of CRE bacteraemia, with a median (interquartile range [IQR]) interval of 25 (range: 0–176) days between episodes. Of these six patients, two had two distinct CRE isolates cultured on the same day, while the remaining four developed a second episode more than 21 days apart. The same organism was isolated in three of the four recurrent episodes. Therefore, in total, 94 episodes of CRE bacteraemia were recorded.

### Characteristics of cases diagnosed with carbapenem-resistant Enterobacterales bacteraemia

The clinical characteristics of cases are described in Table 1. Notably, most cases (79.5%, *n* = 70/88) had one or more comorbid conditions, with 53.4% (*n* = 47/88) of cases diagnosed with an underlying malignancy. HIV status was documented for only 67% (*n* = 59/88) of cases. Other comorbidities not initially listed were documented in 20.5% (*n* = 18/88). These included bronchiectasis and chronic obstructive pulmonary disease, chronic liver disease, dyslipidaemia, hypothyroidism, systemic lupus erythematosus, obesity, chronic pancreatitis, epilepsy and gout. Exposure to immunosuppressive therapy was documented in 56.8% (*n* = 50/88) of cases. Antibiotic exposure in the 6 months prior to CRE bacteraemia was widespread, with 90.9% (*n* = 80/88) of cases having a documented history of prior antibiotic use.

**TABLE 1:** Demographic features and clinical characteristics of cases with carbapenem-resistant Enterobacterales bacteraemia treated in adult wards at Universitas Academic Hospital over a 5-year period (*N* = 88).

Variable	*n*	%
**Age (years)**		
13–29	25	28.4
30–49	24	27.3
50–59	21	23.9
> 60	18	20.4
**Sex**		
Male	35	39.8
Female	53	60.2
**Underlying malignancy**		
Haematological	37	42.0
Solid tumours	10	11.4
None reported	41	46.6
**HIV status**		
Positive	17	19.3
Negative	42	47.7
Unknown	29	33.0
**Underlying comorbidities**		
Acute kidney injury	54	61.4
Hypertension	30	34.1
Cardiovascular disease	17	19.3
Chronic kidney disease	14	15.9
Diabetes	12	13.6
Other	18	20.5
None documented	18	20.5
**Exposure to immunosuppressive therapy**		
Yes	50	56.8
No	38	43.2
**Documented exposure to antibiotics in the past 6 months**		
Yes	80	90.9
No	8	9.1
**Surgical procedure during current admission**		
Yes	36	40.9
No	52	59.1
**Documented hospitalisation in the last 90 days**		
Yes	66	75.0
No	22	25.0

The median length of hospital stay at the time of the positive blood culture was 15 days (IQR: 0–148).

### Logistical data

**FIGURE 1:**
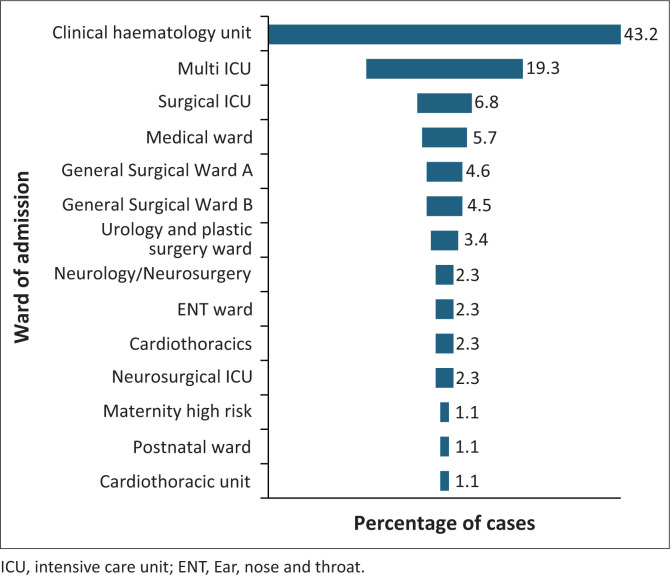
Percentage of cases with carbapenem-resistant Enterobacterales bacteraemia according to ward of admission (*N* = 88).

**FIGURE 2:**
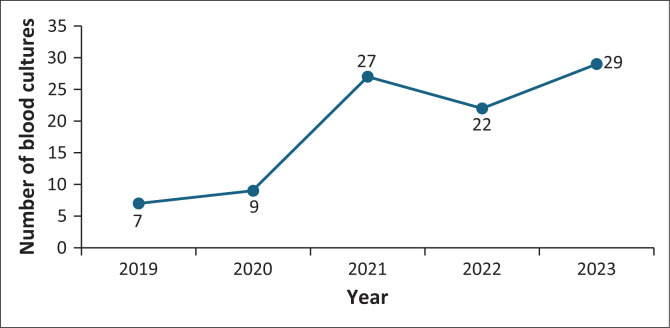
Number of blood cultures per year positive for carbapenem-resistant Enterobacterales in adult wards (*N* = 94).

Most cases were reported from the Clinical Haematology Unit, followed by the multi-disciplinary and surgical intensive care units (ICUs) (Figure 1). A trend towards an increase in the number of reported CRE bacteraemia cases was seen from 2019 to 2020 onwards (Figure 2).

### Patient outcomes

The in-hospital mortality rate among cases with CRE bacteraemia was 56.8% (*n* = 50/88). Exposure to immunosuppressive therapy was associated with a statistically significant increase in in-hospital mortality, with 68% (*n* = 34/50) of deceased cases having a documented recent exposure (odds ratio [OR]: 2.92; 95% confidence interval [CI]: 1.22–7.02). The median length of hospital stay was 26 days (IQR: 4–191). Other clinical characteristics and outcome associations are summarised in Table 2.

**TABLE 2:** Clinical characteristics associated with in-hospital mortality as an outcome (*N* = 88).

Clinical characteristics	Discharged alive (*n* = 38)		Demised in hospital (*n* = 50)		In-hospital mortality		*p*-value
	*n*	%	*n*	%	OR	95% CI	
**Sex**							
Male	16	45.7	19	54.3	1.19	0.50–2.81	0.6968
Female	22	41.5	31	58.5			
**Underlying malignancy**							
Yes	18	38.3	29	61.7	1.53	0.66–3.59	0.3230
No	20	48.8	21	51.2			
**HIV status**							
Positive	8	47.1	9	52.9	1.13	0.36–3.48	0.8379
Negative	21	50.0	21	50.0			
**Comorbidities**							
Yes	28	40.0	42	60.0	1.88	0.66–5.33	0.2386
No	10	55.6	8	44.4			
**Immunosuppressant exposure**							
Yes	16	32.0	34	68.0	2.92	1.22–7.02	0.0165
No	22	57.9	16	42.1			
**Prior antibiotic exposure**							
Yes	33	41.2	47	58.8	2.37	0.53–10.63	0.2583
No	5	62.5	3	37.5			
**Undergone a surgical procedure**							
Yes	16	44.4	20	55.6	0.92	0.39–2.16	0.8423
No	22	42.3	30	57.7			
**Prior hospitalisation**							
Yes	30	46.2	35	53.8	0.62	0.23–1.67	0.3461
No	8	34.8	15	65.2			
**ICU admission**							
Yes	8	32.0	17	68.0	1.93	0.73–5.12	0.1856
No	30	47.6	33	52.4			

OR, odds ratio; CI, confidence interval; ICU, intensive care unit.

### Characteristics of organisms

**FIGURE 3:**
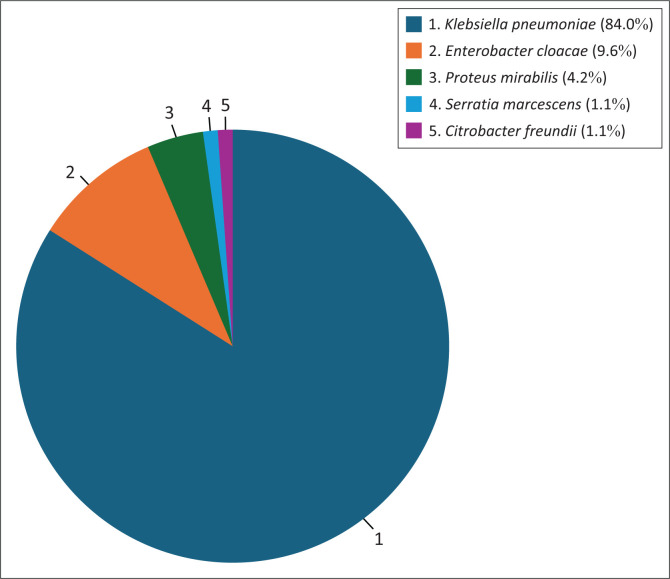
Percentage (%) of organisms cultured (*N* = 94).

Among the 94 episodes of CRE bacteraemia, 84% (*n* = 79/94) of cultures were positive for *Klebsiella pneumoniae*, followed by *Enterobacter cloacae* at 9.6% (*n* = 9/94) and *Proteus mirabilis* with 4.3% (*n* = 4/94). Other organisms accounted for less than 5% of the total burden (Figure 3).

**FIGURE 4:**
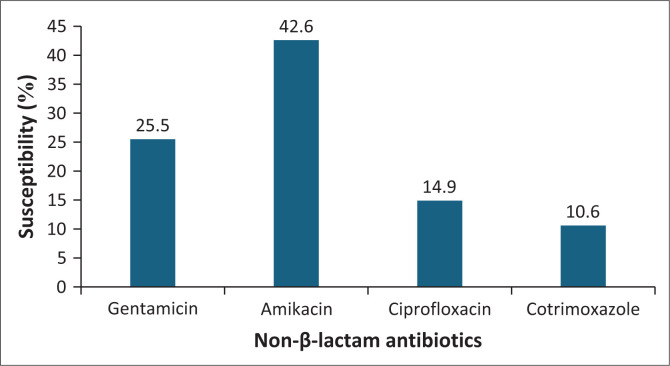
Susceptibility (%) of non-β-lactam antibiotics (*N* = 94).

Figure 4 illustrates the antibiotic susceptibility patterns of the isolates to non-β-lactam antibiotics. Polymyxin susceptibility data were not available as broth micro-dilution was not performed locally during the study period. Carbapenem MICs were not available for this study; however, based on VITEK2 system automated antimicrobial susceptibility testing, ertapenem was reported as susceptible in 3.2% (*n* = 3/94), imipenem in 16% (*n* = 15/94) and meropenem in 30.9% (*n* = 29/94) of isolates.

Carbapenemase genetic testing was performed on 78.7% (*n* = 74/94) of the isolates. Among these, the majority were confirmed to be carbapenemase-producing Enterobacterales (CPE), with carbapenemase genes detected in most isolates. Only 10.8% (*n* = 8/74) of isolates lacked carbapenemase genes, indicating the presence of alternative resistance mechanisms or less common enzymes not included in the test. Notably, four of these eight isolates were identified as *Enterobacter cloacae*. Table 3 summarises the enzymes found in our isolates.

**TABLE 3:** Carbapenemase genotypic results (*N* = 74).

Type of *carbapenemase*	*n*	%
OXA-48	58	78.4
NDM	5	6.7
OXA-48 and NDM	2	2.7
OXA-48, NDM, VIM and KPC	1	1.4
NONE	8	10.8

KPC, *Klebsiella pneumoniae* carbapenemase; NDM, New Delhi metallo-β-lactamase; OXA-48, oxacillinase-48; GES, Guiana extended-spectrum β-lactamase; VIM, Verona integron-encoded metallo-β-lactamase.

### Concurrent sites of isolation

In 50% (*n* = 47/94) of the CRE episodes, an isolated bacteraemia was present. In the remaining episodes, the same CRE organism was isolated from an additional site. The most common additional site was the urine, accounting for 38.3% (*n* = 18/47) of episodes. This was followed by sputum at 23.4% (*n* = 11/47) and tracheal aspirates at 12.8% (*n* = 6/47). Other sites included pus aspirates at 12.8% (*n* = 6/47), line tip cultures at 10.6% (*n* = 5/47), peritoneal fluid at 6.4% (*n* = 3/47) and tissue cultures at 2.1% (*n* = 1/47).

### Antibiotic treatment regimens

Only 91.5% (*n* = 86/94) of CRE episodes were treated with an antibiotic regimen assumed to be effective. Eight episodes were treated with empirical antibiotics that were considered ineffective for CRE. Carbapenem monotherapy was prescribed in 31.9% (*n* = 30/94) of episodes, and a carbapenem with colistin was prescribed in 50% (*n* = 47/94) of episodes. For nine infectious episodes, eight of which had OXA-48 enzyme production confirmed, patients were changed from a carbapenem-containing regimen to ceftazidime–avibactam once the genotypic results became available.

## Discussion

This study aims to investigate the epidemiological and clinical characteristics, as well as the outcomes of patients diagnosed with CRE bacteraemia at Universitas Academic Hospital over a 5-year period. The data revealed important insights into the demographics, comorbidities, clinical areas where infections were reported, microbiological characteristics of CRE infections, as well as factors associated with poor outcomes.

The median age of patients in this study was 46 years, with a higher prevalence of female patients (60.2%). This finding contradicts previous studies, which have noted a higher incidence of CRE infections in male patients, as reported by two recent South African studies that found a prevalence of around 54% in male patients across multiple centres.^6,10^ Several large international multicentre studies have also shown similar trends of male predominance.^11,12,13^ No association between sex and in-hospital mortality was found in this study.

A significant proportion of cases (79.5%) had one or more comorbidities, with hypertension, cardiovascular disease and diabetes being the most common. These findings are in line with previous local and international research, which has shown that CRE infections disproportionately affect patients with multiple comorbid conditions.^6,10,11,12,14^ More than half of the cases (53.4%) had an underlying malignancy, predominantly haematological malignancies. Research indicates that this patient group is at an elevated risk for bloodstream infections because of factors such as immunocompromised state, prolonged neutropenia, high-dose chemotherapy and mucosal barrier damage.^15^ Furthermore, studies have shown a CRE colonisation rate of approximately 10% in rectal swabs from these patients.^11,15^ In April 2023, the final year of the study period, an outbreak of CRE OXA-48 *Klebsiella pneumoniae* occurred in the haematology ward, with five cases identified. Only two cases were bacteraemic and thus included in the study. This selection is unlikely to have biased the results. Although most cases with CRE bacteraemia had an underlying malignancy, suggesting that this group was disproportionately affected, it was not found to be statistically associated with an increased risk of mortality.

Sepsis-related acute kidney injury (AKI) is reported to affect 10% – 67% of patients.^16^ Renal impairment – either AKI, chronic kidney disease (CKD) or acute-on-chronic kidney injury – was prevalent in 62.5% of cases in this study, with 61.4% exhibiting a component of AKI. This highlights the significant burden of AKI in CRE bacteraemia. The risk may be further exacerbated by the use of potential nephrotoxic antibiotics such as amikacin and colistin.^17^ In the literature, there is a paucity of data on AKI that pertains specifically to CRE bacteraemia. The presence of AKI or CKD did not show a statistically significant association with in-hospital mortality.

Immunosuppressive therapy is known to increase the risk of infections with CRE.^11,18^ This study found that 56.8% of patients had been exposed to immunosuppressive therapy that included corticosteroids, chemotherapeutic agents and biologics. Exposure to these agents had a statistically significant association with mortality (*p* = 0.0165), further underscoring the vulnerability of immunocompromised patients to these multidrug-resistant infections.

In our study, the prevalence of HIV infection among patients with a known status was 28.8%, notably higher than the 19.1% reported in the 2022 South African National HIV Prevalence Study for the Free State province.^19^ This elevated prevalence may be partly attributed to the relatively small sample size and the specific subset of tertiary care patients included in the study. In addition to this, people living with HIV (PLHIV) are at an increased risk of colonisation and infection with drug-resistant Enterobacterales because of alterations in their gut microbiome, more frequent exposure to healthcare services and more widespread use of antibiotics in this population.^18^ There was no statistically significant correlation between HIV status and in-hospital mortality, which aligns with findings from a recent epidemiological study conducted on CRE bacteraemia patients in South Africa.^6^ Despite the high HIV prevalence found in this study, the absence of increased mortality may be attributed to the high uptake of antiretroviral therapy (ART) in South Africa, coupled with the fact that most PLHIV are virologically suppressed.^19^ Further data are required to distinguish both the risk of infection and the impact on mortality in virologically unsuppressed patients. The impact of HIV on mortality is often under-reported in large international studies, likely because of the lower prevalence of HIV in those settings, contributing to the paucity in data.

A previous study done in the United States demonstrated that patients with CRE infections had more than 12 times the odds of prior antibiotic use compared to a control group.^20^ A similar tendency may be suggested in our study, with antibiotic exposure in the 6 months prior to the diagnosis of CRE bacteraemia being common, observed in 90.9% of cases. However, it was not associated with in-hospital mortality. This reflects the widespread use of antibiotics in our setting, which exerts selective pressure that contributes to the emergence of resistant organisms. In contrast, prior antibiotic exposure was lower in a recent national study, at 61%.^6^ This discrepancy may be because of the high intensity of care required in our setting, which delivers tertiary care and a specialised haematology service. A large proportion of participants (75%) were re-admitted, which increases environmental exposure to pathogens and the need for empiric nosocomial coverage based on clinical suspicion of infection. Most cases were admitted to the Clinical Haematology Unit, where antibiotic exposure is particularly high because of the management of conditions like neutropenic fever, which often necessitates empiric antibiotic therapy as part of initial emergency management. It is well documented that antibiotic exposure increases the risk of CRE infections compared to carbapenem-sensitive Enterobacterales infections.^11,20^ The data obtained in this study stress the need for strengthening antimicrobial stewardship programmes.

Microbiological analyses revealed that the majority of CRE bacteraemia episodes were caused by *Klebsiella pneumoniae* (84%), followed by *Enterobacter cloacae* (9.6%) and *Proteus mirabilis* (4.3%). The predominance of *Klebsiella pneumoniae* is in keeping with global trends seen across various South African, Asian, European and American studies.^6,10,12,13,14,21,22^ Carbapenemase genetic studies performed on 78.7% of isolates confirmed that most of the CRE organisms were CPE. The predominant carbapenemase was OXA-48 (78.4%), followed by NDM (6.7%). This finding is comparable to other studies done in Europe, Africa, the Middle East and South Africa, where OXA-48 has emerged as the most prevalent carbapenemase.^6,23,24^ These results underline the importance of monitoring carbapenemase production, as it is crucial for determining the appropriate treatment options for CRE infections. Identification of CPE is also essential for prognostication, as it may be associated with worsened outcomes and higher pathogenicity.^6^ Early identification of CRE and CPE strains can potentially assist in the institution of appropriate isolation and IPC measures to curb the spread of these organisms.

In half of the episodes (50%), the only documented site of infection was an isolated bacteraemia. This is concerning, as it complicates efforts to target specific sites for infection control and prevention. Potential source identification was particularly limited in patients with an underlying haematological malignancy, as only 35.1% had the same CRE organism isolated from a concurrent site despite strict protocols guiding microbiological sampling in this patient population. This may reflect a higher prevalence of primary bacteraemia in this population, often related to bacterial gut translocation and may have contributed to skewing of our overall data. In episodes where a concurrent site of isolation with the same CRE organism was confirmed, the most common sample type was the urine (38.3%), followed by sputum (23.4%) and tracheal aspirates (12.8%). While this retrospective study lacked the necessary clinical data to confirm these as sources of infection, this distribution underscores the significant role of the urinary tract and respiratory system as potential sources of CRE bacteraemia, which aligns with findings from other studies.^11,12^ The urinary tract as a potential source highlights the importance of emphasising sterile technique and improving staff training to prevent catheter-associated CRE infections. The significant number of episodes of isolated bacteraemia further emphasises the need for healthcare staff to be educated and trained to actively seek out potential sources of infection. Prompt implementation of source control measures, such as changing catheters and removing or replacing contaminated lines, is essential in reducing the incidence of CRE bacteraemia.

At 56.8%, the in-hospital mortality rate for our patient population with CRE bacteraemia was alarmingly high. Previous reports indicate that CRE infections are associated with high mortality, especially in older patients with underlying comorbidities, prior antibiotic use, surgery during admission, indwelling devices, ICU admission, immunocompromised state and those requiring prolonged hospital stays.^6,10,13,14,20^ We found that our mortality rate was higher than multiple previous local and international studies, where a mortality rate of 31% – 50% is reported.^6,10,12,13,14,22^ The latest national update in 2020 reported a 20.2% lower mortality rate than that found in our study.^6^ This may be multifactorial, with contributing considerations being a population of severely ill tertiary care patients, many of whom had an underlying haematological malignancy, lack of access to rapid diagnostic techniques and limited access to first-line antibiotic regimens for CRE infection during the study period.

Although current international guidelines recommend ceftazidime–avibactam as first-line therapy for OXA-48-producing CRE, this agent was only introduced into local practice from April 2023. As a result, a mere 13.8% (*n* = 8/58) of episodes with a documented OXA-48 enzyme received recommended first-line therapy during the study period. Ceftazidime–avibactam has been shown to be superior to colistin, with significantly lower 30-day mortality rates, emphasising the impact of delayed access to optimal therapy.^25,26^ Furthermore, none of the episodes with NDM or dual NDM and OXA-48 genotypes received the recommended combination of ceftazidime–avibactam plus aztreonam. Instead, the vast majority were managed with second-line regimens, including carbapenem monotherapy (31.9%) and carbapenem plus colistin (50%). Polymyxin B, preferred over colistin because of its more favourable renal toxicity profile, was not available for any of the cases in this cohort.^17^ Alarmingly, 8.5% (*n* = 8/94) of episodes received no antimicrobial regimen active against CRE pathogens. These findings underscore the urgent need for strengthened sepsis surveillance systems, timely molecular diagnostics and improved access to appropriate empiric and targeted antimicrobial therapies in low-resource settings.

There was a noticeable upward trend in infections starting from 2019 to 2020 onwards in our study. The lower infection rates observed prior to 2021 may be attributed to the enhanced IPC measures implemented during the coronavirus disease 2019 (COVID-19) pandemic, including increased use of personal protective equipment, hand hygiene and patient isolation protocols.

### Strengths and limitations

One of the key strengths of this study is its longitudinal design, which spans a 5-year period from 2019 to 2023, allowing for the observation of trends in CRE infections over time. Given the under-representation of the Free State in national studies, these findings are particularly important as they provide critical contextual insights that could inform local healthcare strategies and guide clinicians in managing these infections more effectively. Furthermore, the study employed a comprehensive methodology, ensuring that a wide range of clinical and demographic data were thoroughly documented and analysed, strengthening the overall reliability and validity of the study’s findings.

While this study provides valuable insights, there are limitations to consider. The study’s retrospective design could lead to gaps or inaccuracies in the information that may affect the overall analysis. The local laboratory employs selective reporting as part of its antimicrobial stewardship strategy; consequently, specific MIC data were not available in our study. As a result, susceptibility to certain antimicrobial agents may be under-reported in this cohort. While this research contributes to regional data, its single-centre design, small sample size and specific tertiary patient population may limit generalisability to other settings. Finally, the lack of follow-up after discharge could also impact the ability to assess long-term outcomes of CRE infections.

## Conclusion

This study reveals an alarmingly high in-hospital mortality rate among patients with CRE bacteraemia in our setting, exceeding both national and international averages. Immunosuppressive therapy disproportionately affected individuals, highlighting vulnerable subgroups in urgent need of targeted interventions. These data highlight the need for improved IPC efforts, rapid diagnostics and laboratory support, as well as advocacy for access to more effective antibiotic regimens in our setting. The frequent exposure to prior antibiotics observed in our study further underscores the critical need for robust antimicrobial stewardship to curb the development and spread of resistance. This study contributes valuable insight into a largely understudied population, and these findings can inform local clinical practice, guide public health policy, support advocacy efforts and serve as a foundation for future multicentre studies.

## Appendix 1

### Definition of measurable variables as per original protocol

**Underlying Malignancy:** All patients who had a documented malignancy upon current admission or were undergoing treatment for a malignancy were defined as having an underlying malignancy. Patients who had completed treatment and were documented to be in remission were not considered to have an underlying malignancy.

**Haematological Malignancy:** Defined as any patient admitted to the Haematology unit at Universitas Hospital or treated by the Department of Haematology for a primary neoplastic condition of lymphoid or haematopoietic tissues.

**Human Immunodeficiency Virus (HIV) Status:** HIV positivity was defined as any patient who was documented to be HIV positive as per clinical notes or had a positive HIV test documented on NHLS Labtrak.

HIV negativity was defined as any patient who was documented to be HIV negative based on clinical notes, or in whom an HIV test documented on NHLS Labtrak done in the preceding 12 months was negative.

HIV Status Unknown was defined as any patient with no documentation of HIV status/test results or testing performed more than 12 months prior to study inclusion.

**Hypertension:** Defined as any patient documented as a known hypertensive patient or any patient who was on anti-hypertensive medications based on their pharmacy records.

**Diabetes Mellitus:** A diagnosis of diabetes was recorded for any patient documented in the clinical notes as a known diabetic patient and patients with pharmacy records indicating the use of oral hypoglycemic agents or insulin.

**Acute Renal Impairment:** Acute renal impairment was defined as any patient who had documented acute renal impairment based on clinical notes. Laboratory data that indicated an increase in serum creatinine of more or equal to 1.5 times the baseline, or an increase of more than 26.5 µmol/L during the admission in which the CRE bacteremia occurred, was also documented to have acute renal impairment.

**Chronic Renal Impairment:** Chronic renal impairment was defined as any patient documented to have chronic renal impairment based on clinical notes. Patients who had an estimated glomerular filtration rate of less than 60 mL/min/1.73m^2^ for at least two readings three months apart were included in this group.

**Cardiovascular Disease:** Any patient with a documented stroke, peripheral vascular disease, or myocardial infarction as well as any patient documented to have arrhythmia, heart failure, or valvular heart disease was defined as having cardiovascular disease.

**Recent Exposure to Immunosuppressive Therapy:** Documented exposure to immunosuppressive therapy in the preceding three months prior to documentation with CRE bacteremia. Immunosuppressive therapy was defined as clinical notes or pharmacy records indicating exposure to the following agents: cyclophosphamide, azathioprine, cyclosporine, tacrolimus, mycophenolate mofetil, and rituximab.

**Antibiotic Use Within the Last Six Months:** This included any patient documented to have self-reported antibiotic use in the clinical notes or in whom antibiotic use was confirmed by hospital pharmacy records within the six months preceding admission.

## References

[1] Naghavi M, Vollset SE, Ikuta KS, et al. Global burden of bacterial antimicrobial resistance 1990–2021: A systematic analysis with forecasts to 2050. Lancet. 2024;404(10459):1199–1226. 10.1016/S0140-6736(24)01867-1 39299261 PMC11718157

[2] Cai Y, Hoo GSR, Lee W, et al. Estimating the economic cost of carbapenem resistant Enterobacterales healthcare associated infections in Singapore acute-care hospitals. PLoS Glob Public Health. 2022;2(12):e0001311. 10.1371/journal.pgph.0001311 36962882 PMC10021918

[3] Murray CJ, Ikuta KS, Sharara F, et al. Global burden of bacterial antimicrobial resistance in 2019: A systematic analysis. Lancet. 2022;399(10325):629–655. 10.1016/S0140-6736(21)02724-0 35065702 PMC8841637

[4] Suay-García B, Pérez-Gracia MT. Present and future of carbapenem-resistant *Enterobacteriaceae* (CRE) infections. Antibiotics (Basel). 2019;8(3):122. 10.3390/antibiotics8030122 31430964 PMC6784177

[5] Tubb CM, Tubb M, Hooijer J, Chomba R, Nel J. Carbapenem-resistant Enterobacterales (CRE) colonisation as a predictor for subsequent CRE infection: A retrospective surveillance study. S Afr J Infect Dis. 2025;40(1):a687. 10.4102/sajid.v40i1.687 PMC11830835 39968233

[6] Lowe M, Shuping L, Perovic O. Carbapenem-resistant Enterobacterales in patients with bacteraemia at tertiary academic hospitals in South Africa, 2019-2020: An update. S Afr Med J. 2022;112(8):545–552. 10.7196/SAMJ.2022.v112i8.16351 36214398

[7] Rabaan AA, Eljaaly K, Alhumaid S, et al. An overview on phenotypic and genotypic characterisation of Carbapenem-resistant *Enterobacterales*. Medicina. 2022;58(11): 1675. 10.3390/medicina58111675 36422214 PMC9696003

[8] Centers for Disease Control and Prevention. Carbepenem resistant Enterobacterales [homepage on the Internet]. [cited 2024 Feb 06]. Available from: https://www.cdc.gov/hai/organisms/cre/index.html

[9] Xu L, Sun X, Ma X. Systematic review and meta-analysis of mortality of patients infected with carbapenem-resistant *Klebsiella pneumoniae*. Ann Clin Microbiol Antimicrob. 2017;16(1):1–12. 10.1186/s12941-017-0191-3 28356109 PMC5371217

[10] Mbele S, Vasaikar S. Risk factors for Carbapenem-resistant *Enterobacterales* infections: A case-control study. S Afr Fam Pract. 2025;67(1):a6029. 10.4102/safp.v67i1.6029 PMC11886460 39935157

[11] Pérez-Galera S, Bravo-Ferrer JM, Paniagua M, et al. Risk factors for infections caused by carbapenem-resistant Enterobacterales: An international matched case-control-control study (EURECA). EClinicalMedicine. 2023;57:101871. 10.1016/j.eclinm.2023.101871 36895801 PMC9989660

[12] Van Duin D, Arias CA, Komarow L, et al. Molecular and clinical epidemiology of carbapenem-resistant Enterobacterales in the USA (CRACKLE-2): A prospective cohort study. Lancet Infect Dis. 2020;20(6):731–741. 10.1016/S1473-3099(19)30755-8 32151332 PMC7473597

[13] Zhou C, Jin L, Wang Q, et al. Bloodstream infections caused by carbapenem-resistant *Enterobacterales*: Risk factors for mortality, antimicrobial therapy and treatment outcomes from a prospective multicenter study. Infect Drug Resist. 2021;14:731–742. 10.2147/IDR.S294282 33658810 PMC7917342

[14] Chen L, Han X, Li Y, Li M. Assessment of mortality-related risk factors and effective antimicrobial regimens for treatment of bloodstream infections caused by carbapenem-resistant *Enterobacterales*. Antimicrob Agents Chemother. 2021;65(9):e0069821. 10.1128/aac.00698-21 34228539 PMC8370219

[15] Chen X, Wen X, Jiang Z, Yan Q. Prevalence and factors associated with carbapenem-resistant Enterobacterales (CRE) infection among hematological malignancies patients with CRE intestinal colonization. Ann Clin Microbiol Antimicrob. 2023;22(1):3. 10.1186/s12941-023-00554-6 36627626 PMC9832636

[16] Manrique-Caballero CL, Del Rio-Pertuz G, Gomez H. Sepsis-associated acute kidney injury. Crit Care Clin. 2021;37(2):279–301. 10.1016/j.ccc.2020.11.010 33752856 PMC7995616

[17] Lasko MJ, Nicolau DP. Carbapenem-resistant Enterobacterales: Considerations for treatment in the era of new antimicrobials and evolving enzymology. Curr Infect Dis Rep. 2020;22(6):1–12. 10.1007/s11908-020-0716-3 32034524 PMC7223591

[18] Henderson HI, Ruegsegger L, Alby K, et al. Antimicrobial-resistant Enterobacterales colonization in people with HIV. JAC Antimicrob Resist. 2022;4(4):dlac082. 10.1093/jacamr/dlac082 35935279 PMC9345307

[19] Human Sciences Research Council. The sixth South African National HIV prevalence, incidence, behaviour and communication survey [homepage on the Internet]. Pretoria: HSRC; 2022 [cited 2024 Feb 06]. Available from: https://www.dsti.gov.za/index.php/media-room/latest-news/3567-6th-south-african-hiv-prevalence-incidence-behaviour-and-communication-survey

[20] Stuever DM, Ferketich AK, Lee J, Stevenson KB, Wittum TE. Case-case-control study of risk factors for carbapenem-resistant Enterobacterales infections among hospitalized patients. Antimicrob Steward Healthc Epidemiol. 2022;2(1):e118. 10.1017/ash.2022.244 36483348 PMC9726559

[21] Barros A, Monroy H, Bergo P, Beck E, David L, Rigatto MH. Antimicrobial stewardship programme associated with earlier prescription of in vitro susceptible therapy and lower 14-day mortality in patients with carbapenem-resistant Enterobacterales bacteraemia: A cohort study. J Glob Antimicrob Resist. 2022;28:130–135. 10.1016/j.jgar.2021.12.011 34933141

[22] So-Ngern A, Osaithai N, Meesing A, Chumpangern W. Mortality rate and factors associated with mortality of carbapenem-resistant Enterobacteriaceae infection. Drug Target Insights. 2023;17:120–125. 10.33393/dti.2023.2622 38028024 PMC10630699

[23] Boyd SE, Holmes A, Peck R, Livermore DM, Hope W. OXA-48-like β-Lactamases: Global epidemiology, treatment options, and development pipeline. Antimicrob Agents Chemother. 2022;66(8):e00216-22. 10.1128/aac.00216-22 35856662 PMC9380527

[24] Karlowsky JA, Bouchillon SK, El Mahdy Kotb R, Mohamed N, Stone GG, Sahm DF. Carbapenem-resistant Enterobacterales and *Pseudomonas aeruginosa* causing infection in Africa and the Middle East: A surveillance study from the ATLAS programme (2018-20). JAC Antimicrob Resist. 2022;4(3):dlac060. 10.1093/jacamr/dlac060 35733913 PMC9204471

[25] Chen Y, Huang HB, Peng JM, Weng L, Du B. Efficacy and safety of Ceftazidime-Avibactam for the treatment of Carbapenem-resistant *Enterobacterales* bloodstream infection: A systematic review and meta-analysis. Microbiol Spectr. 2022;10(2):e02603-21. 10.1128/spectrum.02603-21 35377233 PMC9045088

[26] Chen J, Hu Q, Zhou P, Deng S. Ceftazidime–avibactam versus polymyxins in treating patients with carbapenem-resistant Enterobacteriaceae infections: A systematic review and meta-analysis. Infection. 2024;52(1):19–28. 10.1007/s15010-023-02108-6 37878197 PMC10810944

